# Aggressive angiomyxoma of the pelvis and abdominal wall: Dramatic response to chemical ablation therapy

**DOI:** 10.3389/fonc.2023.1154283

**Published:** 2023-03-16

**Authors:** Liu Li, He Chuang, Liu He-Nan, Li Dong-Yuan, Liang Qing-Hua, Li Wei, Li Liang-Shan, Li Ting-Yuan, Huang Xue-Quan

**Affiliations:** Department of Nuclear Medicine (Treatment Center of Minimally Invasive Intervention and Radioactive Particles), First Affiliated Hospital of the Army Medical University, Chongqing, China

**Keywords:** aggressive angiomyxoma, operation, chemical ablation, therapy, CT-guided

## Abstract

**Objective:**

Aggressive angiomyxoma (AAM) is a rare, locally aggressive soft tissue neoplasm with a marked tendency for local recurrence after surgery. Although hormone therapy, radiation therapy, and vascular embolization can be performed, we investigated the safety and efficacy of a new chemical ablation protocol for AAM.

**Methods:**

This study included two female AAM patients from 2012 to 2016. The patients’ clinical and imaging data were collected. The amount of anhydrous ethanol and glacial acetic acid used for chemical ablation was documented, and the management of any complications was detailed.

**Results:**

The maximum dimensions of the residual tumor were 12.6 cm and 14.0 cm. In one case, the lesion was in the pelvis and protruded into the vulva. Eighty milliliters of liquid with a mixture of glacial acetic acid, anhydrous ethanol, and iohexol (10:9:1) was used for chemical ablation therapy *via* multipoint injections with a single needle. However, a pelvic fistula developed 1 month later. In another case, the lesion was located in the abdominal wall. The ablation procedure was improved by performing chemical ablation therapy with multiple needles for multi-point injections of smaller than 30 ml injections for each procedure. To date, no recurrence or metastasis has been observed in the two cases.

**Conclusion:**

The preferred treatment for AAM is complete resection. Chemical ablation therapy is a novel adjuvant therapy for AMM. Nonetheless, more research is needed to confirm these findings.

## Introduction

AAM was first reported in 1983 (1). AAM is more common in women but can also occur in men, and the peak incidence is between 31 and 35 years old. These lesions mainly occur in the female vulva, perineal body, vaginal wall, groin, pelvic cavity, retroperitoneal area, and bladder. Resection remains the mainstay of treatment for AAM; however, the recurrence rate within three years postoperatively is approximately 70% (2).

Radiotherapy, GnRH therapy, and other approaches have already been reported to address residual tumors (3–5), but the local control rates are poor. We report two cases of residual AAM that were treated with chemical ablation therapy.

## Methods

**Figure 1:**
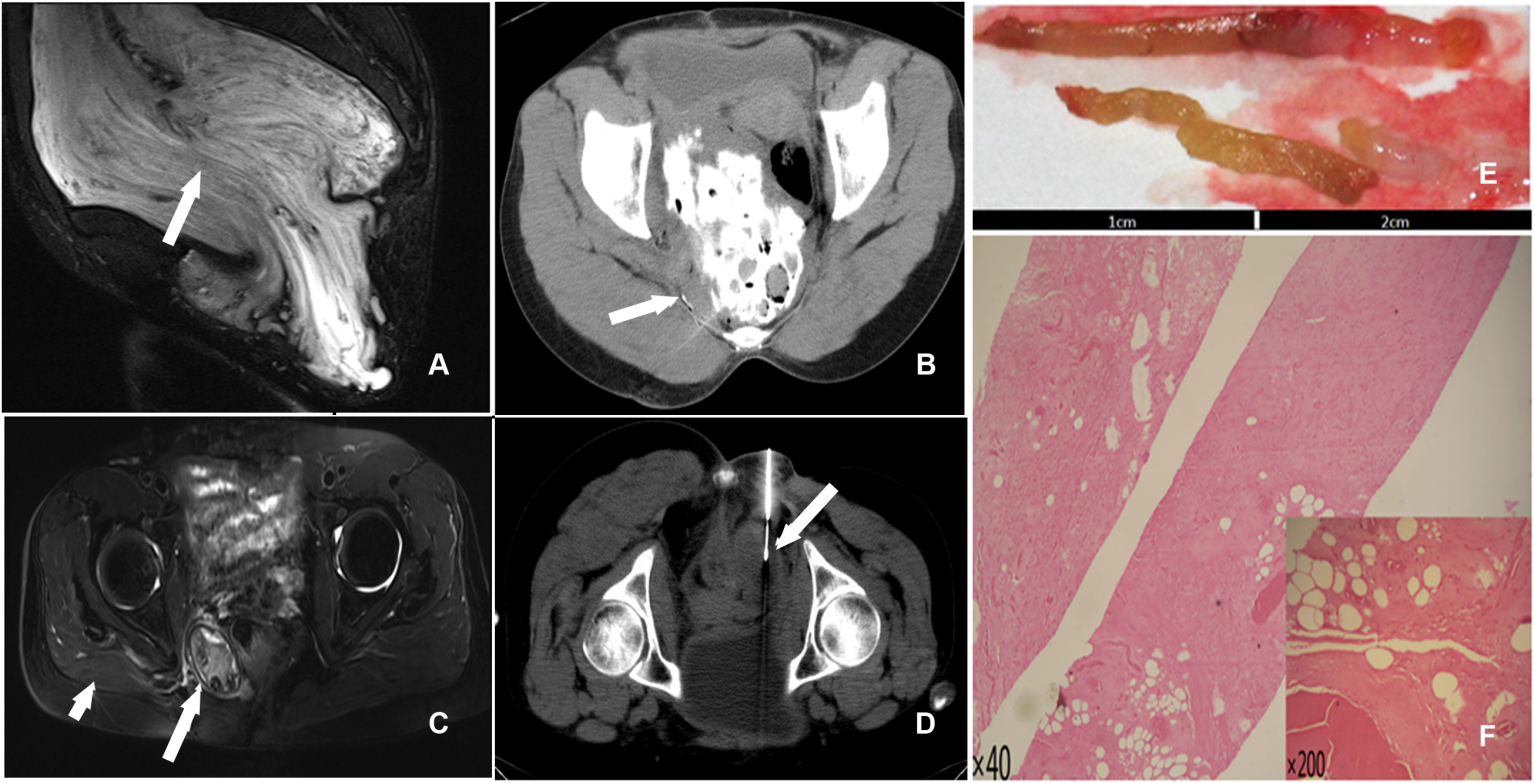
**(A)** T2 STIR (sagittal) scan showed a huge pelvic mass with long T2 signals and a perineal hernia; a swirl sign, a feature of aggressive angiomyxoma, was seen inside the tumor (arrow). **(B)** The liquid mixture overflowed from the needle track after a percutaneous injection of acetic acid during therapy (arrow). **(C)** T2 STIR (axial) scan exhibited an oval mixed signal on the right side of the pelvic floor that measured approximately 4.98 cm X 3.38 cm. The boundary indicated a distinct peripheral area with circular hypointensity (long arrow). Atrophy of the right gluteus maximus muscles was seen (short arrowhead). **(D)** CT-guided percutaneous needle biopsy of the pelvic mass (arrow). **(E)** The gross specimen revealed two 2-centimeter brown-yellow tissues. **(F)** Numerous collagen fibers, necrotic tissue, and hyaline degeneration tissue were seen in the tissue (HE40, HE200).

The first case involved a 48-year-old woman with a right pelvic mass but no redness, swelling, or tenderness. Magnetic resonance imaging (MRI) of the pelvis revealed a significant abdominopelvic focus with a perineal hernia (**Figure 1A**), with a lesion size of 28.0 cm×20.0 cm×10.0 cm. Histological examination revealed spindle-shaped and stellate-shaped cells scattered in a background of loose myxoid stoma with numerous blood vessels of varying caliber. Immunohistochemistry revealed that the cells were positive for estrogen receptor (ER), progesterone receptor (PR), vimentin, desmin, and CD34. The volume of residual disease after excision was 571.8 cm^3^ (the residual tumor diameter was 12.6 cm). [Calculated by volume measurement software of the CT machine (machine type: SOMATO plus 4; scanning parameters: 120 kV, 130 mA; layer thickness: 5 mm) by adding up all the area, then multiplying by the thickness of each layer]. CT-guided percutaneous chemical ablation *via* multipoint injections was performed with a single needle. An 80 mL liquid mixture of glacial acetic acid, anhydrous ethanol, and iohexol (10:9:1) was injected initially. Multidirectional percutaneous puncture was performed under CT guidance. Diffusion ranges were defined according to the contrast agent ranges for CT scans (**Figure 1B**). A year later, CT revealed that the residual disease volume was approximately 137.3 cm^3^. The residual diseases were retreated with a 20 mL liquid mixture containing glacial acetic acid, anhydrous ethanol, and iohexol (4:5:1) for chemical ablation.

**Figure 2:**
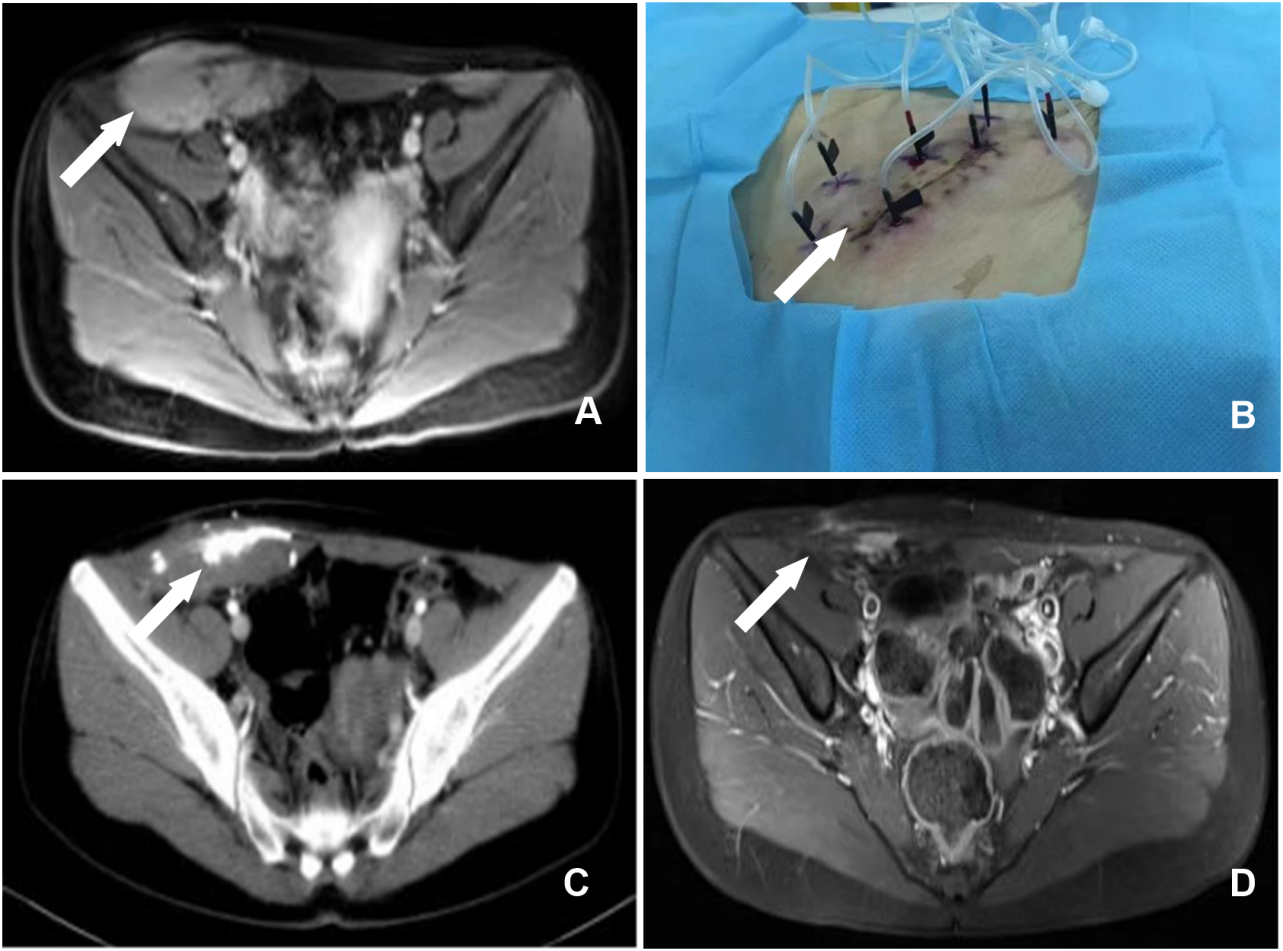
**(A)** A enhanced MRI examination of the pelvis revealed a massive abnormal signal in the lower right abdomen, occupying the rectus abdominis, transverse abdominis, and internal and external oblique muscles (arrow). **(B)** Body surface image showing multipoint injections with multiple needles. **(C)** CT demonstrated the effect of high-density contrast media in the region of the soft tissue mass in the pelvic wall one month after chemical ablation therapy, and the surrounding space was clear (arrow). **(D)** MRI revealed a considerable reduction in the pelvic wall mass 17 months following chemical ablation therapy and a few increased signals during the enhancement phase (arrow).

In another case, a 20-year-old female patient presented with a mass in the right abdominal wall. B-ultrasound, CT, and MRI scans revealed an 8.1 cm x 14.1 cm lesion in the right abdominal wall (**Figure 2A**). A 10.0 cm × 12.0 cm mass was palpable in the lower right abdominal wall intraoperatively. The patient declined total resection for aesthetic reasons. After that, the patient underwent CT-guided percutaneous chemical ablation treatment *via* multipoint injections administered with multiple needles (**Figure 2B**). The tumor volume before ablation was 560.6 cm^3^. Thirty milliliters of liquid containing glacial acetic acid, anhydrous ethanol, and iohexol (1:4:1) was injected initially. One month after the operation, a reexamination found that the tumor volume was 189.4 cm^3^ (**Figure 2C**). Subsequently, the patient underwent three ablations over one year.

The chemotherapy ablation details of the two patients are shown in **Table 1**.

**Table 1:** The chemotherapy ablation details.

Case	Treatment time	Volume(cm3)	Mixture ratio^*^	Total dose(ml)
Case 1	2012/1/12	571.8	10:9:1	80
	2013/2/26	137.3	4:5:1	20
Case 2	2016/8/17	560.6	1:4:1	30
	2016/9/29	189.4	7:27:5	20
	2017/1/10	11.6	3:14:3	20

*glacial acetic acid:anhydrous ethanol:iohexol.

## Results

Postoperatively, persistent lesions in the abdominal wall and pelvic cavity were observed in two female patients. The greatest dimension of the remaining tumor in the first patient was 12.6 cm. The lesion achieved a complete response after two rounds of chemical ablation. There has been no recurrence or metastasis of the lesion during a 10-year follow-up. After surgery, there was locally blue-stained skin, a drop in body temperature, and swelling in the right hip. Intravenous injections of 5% NaHCO_3_ 250 mL and 20% mannitol 125 mL, as well as pain management, were used as treatments. One month later, a right pelvic fistula developed, with purple-thickened mucus spilling from the fistula. After four months of serial dressing changes and furacilin debridement, the fistula healed. Although related complications occurred in this case, the complications could be controlled and did not affect the ablation therapy result. Follow-ups were performed 3.1 years after the first ablation. CT showed that the volume of the residual disease was only 67.4 cm^3^; MR imaging revealed an oval-mixed signal on the right side of the pelvic floor, and no characteristic swirl sign was found. The boundary showed annular hypointensity, and a distinct peripheral space was seen (**Figure 1C**). A second CT-guided percutaneous biopsy was conducted (**Figure 1D**). Postoperative gross specimens showed two 2-centimeter brown-yellow tissues (**Figure 1E**), and HE staining showed a large number of collagen fibers, necrotic tissue, and hyaline degeneration in the tissue (**Figure 1F**).

In another patient, the greatest dimension of the remnant tumor was 14.0 cm. After three rounds of chemical ablation, the lesion achieved a complete response (**Figure 2D**). The lesion has not recurred or metastasized during a 6-year follow-up. The outer skin of the right thigh felt numb on the first day following the procedure. The numbness disappeared on the third day following treatment, and the patient was discharged without any discomfort, skin rupture, or other harmful effects.

## Discussion

The two exceptional cases in this study demonstrated that owing to our technological progress, both AAM cases were controlled for a long time, even though the first case developed a pelvic fistula, but this does not lessen our confidence in employing this strategy.

AAM is a rare, specialized, slow-growing soft tissue tumor that typically spreads locally. AAM primarily affects the pelvic, perineal, and vulvar areas in adult women, particularly those of reproductive age. Before surgery, this disease is not easy to diagnose. AAM presents various symptoms, ranging from an asymptomatic vulvar or perineal nodule to a massive pelvic mass detected on imaging. Because it might resemble a labial, Bartholin’s, or Gartner’s duct cyst, misdiagnosis is to be expected (6). Because of the nature of AAM invasion, radical excision is difficult. Because there is no statistically significant difference in recurrence rate between radical and restricted excision, radical resection may not be the best option (3, 7–11).

Furthermore, hormone treatment, radiotherapy, and vascular embolization therapy have some curative effects on AAM. However, there is some uncertainty in the literature (12–14). As a result, we started an experimental therapy for AMM some years ago.

According to the volume equation V(ml)=4/3(R+0.5)^3^ (15), the volume of acetic acid should be approximately 755.1 mL, which we did not follow. We used a specific quantity of acetic acid and ethanol because the ethanol could not adequately disseminate due to the fiber separation in the lesion. Additionally, acetic acid can compensate for this difference (16). However, the first patient experienced substantial complications, including acute renal failure and pelvic fistula drainage that lasted for four months. These factors are responsible for this outcome: First, the amount of acetic acid-ethanol used for a single injection was substantial; second, the injection pressure of the acetic acid mixture was too high, and the mixture from the needle overflowed, causing injury to normal tissue. However, in the other patient, we modified our technique for repeat therapy to multipoint injections with multiple needles for low-dose chemical ablations. We achieved reasonable local control and no complications.

According to our modified procedure, chemical ablation is a successful treatment for AMM and can be employed as adjuvant therapy. However, due to the low incidence of AAM, no more cases have been available for observational research. It is also challenging to determine the right quantities of absolute ethanol and acetic acid. The critical information we provided can enable other researchers can perform more studies.

In summary, AAM is rare, and CT-guided chemical ablation is a safe and effective method for treating residual AMM. Repeated therapy, multipoint injections with multiple needles and low-dose ablation can also achieve an excellent curative effect and are beneficial for preventing complications.

## Author contributions

LL and HX-Q: study conceptualization and design. LL and HC: analyzed and interpreted data, manuscript preparation, and contributed equally to this paper. LH-N, LD-Y, LQ-H, LW, LL-S, and LT-Y: analyzed and collected data. All authors contributed to the article and approved the submitted version.

## References

[1] Steeper TA Rosai J. Aggressive angiomyxoma of the female pelvis and perineum.Report of nine cases of a distinctive type of gynecologic soft-tissue neoplasm. Am J Surg Pathol (1983) 7(5):463–75. doi: 10.1097/00000478-198307000-00009 6684403

[2] Lee KA Seo JW Yoon NR Lee JW Kim BG Bae DS. Aggressive angiomyxoma of the vulva: A case report. Obstet Gynecol Sci (2014) 57:164–7. doi: 10.5468/ogs.2014.57.2.164 PMC3965702 24678492

[3] Khelifi S Ben Ali A Tagougui W Jaoua H Chammakhi C Chadly A. Perineal recurrence of an aggressive angiomyxoma: Is an incomplete resection useful? J chirurgie (2009) 146:416–8. doi: 10.1016/j.jchir.2009.08.017 19772961

[4] Suleiman M Duc C Ritz S Bieri S. Pelvic excision of large aggressive angiomyxoma in a woman: irradiation for recurrent disease. Int J gynecological Cancer (2006) 16 Suppl 1:356–60. doi: 10.1136/ijgc-00009577-200602001-00063 16515622

[5] Sun NX Li W. Aggressive angiomyxoma of the vulva: case report and literature review. J Int Med Res (2010) 38:1547–52. doi: 10.1177/147323001003800439 20926030

[6] Haldar K Martinek IE Kehoe S. Aggressive angiomyxoma: a case series and literature review. Eur J Surg Oncol (2010) 36:335–9. doi: 10.1016/j.ejso.2009.11.006 19954923

[7] Chan YM Hon E Ngai SW Ng TY Wong LC. Aggressive angiomyxoma in females: is radical resection the only option? Acta obstetricia gynecologica Scandinavica (2000) 79:216–20. doi: 10.1034/j.1600-0412.2000.079003216.x 10716303

[8] Siassi RM Papadopoulos T Matzel KE. Metastasizing aggressive angiomyxoma. New Engl J Med (1999) 341:1772. doi: 10.1056/NEJM199912023412315 10610453

[9] Blandamura S Cruz J Faure VL Machado PI Ninfo V. Aggressive angiomyxoma: a second case of metastasis with patient’s death. Hum Pathol (2003) 34:1072–4. doi: 10.1053/S0046-8177(03)00419-2 14608546

[10] Geng J Cao B Wang L. Aggressive angiomyxoma: an unusual presentation. Korean J Radiol (2012) 13:90–3. doi: 10.3348/kjr.2012.13.1.90 PMC3253408 22247641

[11] Han-Geurts IJ van Geel AN van Doorn L Bakker Md Eggermont AM Verhoef C. Aggressive angiomyxoma: multimodality treatments can avoid mutilating surgery. Eur J Surg Oncol (2006) 32:1217–21. doi: 10.1016/j.ejso.2006.06.008 16870390

[12] Kooy J Carlson V Saciragic L Sawhney S Nelson G. A case series of aggressive angiomyxoma: Using morphologic type and hormonal modification to tailor treatment. Gynecologic Oncol Rep (2021) 36:100765. doi: 10.1016/j.gore.2021.100765 PMC8066423 33912645

[13] Rhomberg W Jasarevic Z Alton1 R Kompatscher P Beer G Breitfellner G. Aggressive angiomyxoma: Irradiation for recurrent disease. Strahlenther Onkol (2000) 176:324–6. doi: 10.1007/s000660050015 10962999

[14] Suleiman M Duc C Ritz S Bieri S. Pelvic excision of large aggressive angiomyxoma in a woman: irradiation for recurrent disease. Int J Gynecol Cancer (2006) 16(Suppl. 1):356–60. doi: 10.1136/ijgc-00009577-200602001-00063 16515622

[15] Shiina S Tagawa K Unuma T Fujino H Uta Y Niwa Y. Percutaneous ethanol injection therapy of hepatocellular carcinoma: analysis of 77 patients. AJR Am J Roentgenol (1990) 155:1221–6. doi: 10.2214/ajr.155.6.2173384 2173384

[16] Hou C Chen SC Chang WY Chen CH. Comparison of necrotic characteristics and benefits between 50% acetic acid and pure ethanol in local hepatic injection: a study in rats. Kaohsiung J Med Sci (1999) 15:414–8. 10465923

